# Can elevated concentrations of ALT and AST predict the risk of ‘recurrence’ of COVID-19?

**DOI:** 10.1017/S0950268820002186

**Published:** 2020-09-21

**Authors:** L. Z. Chen, Z. H. Lin, J. Chen, S. S. Liu, T. Shi, Y. N. Xin

**Affiliations:** 1 Department of Infectious Disease, Qingdao Municipal Hospital Group, Qingdao, China; 2 Department of Gastroenterology, Qingdao Municipal Hospital Group, Qingdao, China; 3 Department of Respiratory and Critical Care Medicine, Tongji Hospital, Tongji Medical College, Huazhong University of Science and Technology, Wuhan, China; 4 Hepatology Laboratory, Qingdao Municipal Hospital Group, Qingdao, China; 5 Digestive Disease Key Laboratory of Qingdao, Qingdao, China; 6 Department of Gastroenterology, Qingdao Municipal Hospital Group, Dalian Medical University, Qingdao, China

**Keywords:** Clinical characteristic, coronavirus disease 2019, predictive factor, recurrence, severe acute respiratory syndrome coronavirus-2

## Abstract

‘Recurrence’ of coronavirus disease 2019 (COVID-19) has triggered numerous discussions of scholars at home and abroad. A total of 44 recurrent cases of COVID-19 and 32 control cases admitted from 11 February to 29 March 2020 to Guanggu Campus of Tongji Hospital affiliated to Tongji Medical College Huazhong University of Science and Technology were enrolled in this study. All the 44 recurrent cases were classified as mild to moderate when the patients were admitted for the second time. The gender and mean age in both cases (recurrent and control) were similar. At least one concomitant disease was observed in 52.27% recurrent cases and 34.38% control cases. The most prevalent comorbidity among them was hypertension. Fever and cough being the most prevalent clinical symptoms in both cases. On comparing both the cases, recurrent cases had markedly elevated concentrations of alanine aminotransferase (ALT) (*P* = 0.020) and aspartate aminotransferase (AST) (*P* = 0.007). Moreover, subgroup analysis showed mild to moderate abnormal concentrations of ALT and AST in recurrent cases. The elevated concentrations of ALT and AST may be recognised as predictive markers for the risk of ‘recurrence’ of COVID-19, which may provide insights into the prevention and control of COVID-19 in the future.

## Introduction

Wuhan Municipal Health Commission, China reported a cluster of pneumonia cases, on 31 December 2019, which was officially named coronavirus disease 2019 (COVID-19) by WHO [1, 2]. Due to its alarming levels of spread and severity of COVID-19, the Director-General of WHO declared the COVID-19 a Public Health Emergency of International Concern on 30 January 2020 and a global pandemic on 11 March 2020, respectively [3, 4]. COVID-19 has emerged to be a fatal disease and caused panic worldwide. A total of 11 468 979 confirmed cases of COVID-19 were reported by WHO by 7 July 2020, which also included 535 181 deaths globally [5]. However, a series of prevention and control steps, as well as precise medical treatment measures, have prevented and controlled the epidemic situation in China and have achieved phased results. China followed the five stages of the arduous course of anti-epidemic: (i) immediate response to the sudden epidemic situation, (ii) initial containment of the epidemic situation, (iii) the number of new cases in China gradually reduced to a single digit, (iv) achieve decisive results in ‘Wuhan and Hubei defense Wars’, (v) normalised national epidemic prevention and control. However, a rise in the number of confirmed cases can be seen abroad, and the task of global epidemic preventive control is still arduous.

Nevertheless, with thorough and increased understanding and accumulation of the epidemiological characteristics, pathological changes, clinical characteristics, diagnosis and treatment of COVID-19, many patients have been discharged from hospital [6]. Another challenge that has attracted wide attention is the recurrence/reactivation/reinfection/relapse/redetectable positive/turned positive/false-negative of COVID-19 and has triggered numerous discussions of scholars at home and abroad. All of the recurrent cases met the discharge criteria according to the diagnosis and treatment protocol for novel coronavirus pneumonia (NCP; trial version 7), which including negative result for nuclei acid tests twice consecutively on respiratory tract specimens such as nasopharyngeal swabs and sputum (24 h sampling interval at least). Recently, the number of recurrent cases has increased, and/or test results of discharged patients of COVID-19 undergoing real-time reverse transcription-polymerase chain reaction (RT-PCR) test turned positive [7–10]. However, the typical clinical characteristics, risk factors or predictive markers, and therapeutic strategies of the recurrent cases remain unclear.

This study aimed to investigate the clinical and laboratory characteristics as well as the potential risk factors or predictive markers of the recurrent cases of COVID-19 to provide certain scientific data for effective prevention and control measures in the future.

## Methods

### Study design and patients

The COVID-19 patients admitted to the Guanggu Campus of Tongji Hospital affiliated to Tongji Medical College Huazhong University of Science and Technology were reviewed from 11 February to 29 March 2020. Dr Yongning Xin and Dr Lizhen Chen were members of the seventh aid medical team in Hubei Province from Shandong Province. A total of 44 recurrent COVID-19 cases, which tested positive for severe acute respiratory syndrome coronavirus-2 (SARS-CoV-2) nucleic acid after 14-day isolation post-discharge were recruited. In addition, 32 control cases matched for sex and age were also randomly recruited for this study. The control cases represent negative SARS-CoV-2 nucleic acid tests after 14-day isolation post-discharge. Based on the diagnosis and treatment protocol for NCP (trial version 7) issued by the National Health Commission & National Administration of Traditional Chinese Medicine, COVID-19 is diagnosed [6]. The discharge criteria for COVID-19 patients are as follows [6]: (1) body temperature to return to normal for more than 3 consecutive days; (2) respiratory symptoms are resolved; (3) pulmonary imaging showing the significantly absorbed lung inflammation; (4) negative result for nuclei acid tests twice consecutively on respiratory tract specimens such as nasopharyngeal swabs and sputum (24 h sampling interval at least).

The Ethics Committee of Qingdao Municipal Hospital (approval number: 2020-040) approved the study protocol. Moreover, this study was conducted in accordance with the principles of the World Medical Association Declaration of Helsinki. Due to the spread and severity of COVID-19, written informed consent was waived.

### Data acquisition

Electronic medical records and nursing records provided all the basic clinical information (such as gender, age, clinical manifestations, comorbidities) and laboratory results of the patients, which were collected by two investigators (Lizhen Chen and Juan Chen). We collected the second admission blood tests of recurrent cases and the before-discharge blood tests of controls.

The laboratory results consisted of NCP virus-specific immunoglobulin G (IgG), NCP virus-specific IgM, inflammatory factors (interleukin (IL)-2R, IL-6, IL-8 and tumour necrosis factor *α* (TNF-*α*)), albumin (ALB, 35–52 g/l), alanine transaminase (ALT, ⩽33 U/l), aspartate transaminase (AST, ⩽32 U/l), alkaline phosphatase (ALP, 35–105 U/l), *γ*-glutamyltransferase (GGT, 6–42 U/l), creatine kinase (CK, ⩽170 U/l), lactate dehydrogenase (LDH, 135–214U /l), blood urea nitrogen (BUN, 3.1–8.8 mmol/l), serum creatinine (Scr, 45–84 μmol/l), blood cell count, coagulation markers, myocardial markers, lymphocyte subsets and so on. Zhonghua Lin and Te Shi checked the dates of collection independently and entered into the database.

### Statistical analysis

All statistical analyses were performed using Statistical Package for the Social Sciences (SPSS), version 22.0, statistical software (SPSS Inc., Chicago, IL, USA). Continuous variables were showed as mean ± standard deviation, and the Student's *t* test or paired-samples *t* test was done. The *χ*^2^ test or Fisher's exact test was used to analyse categorical variables and represented as numbers and percentages. A value of *P* < 0.05 was considered statistically significant.

## Results

### Basic clinical and demographic characteristics of recurrent cases and control cases

A total of 44 recurrent cases of COVID-19 and 32 control cases admitted to the Guanggu Campus of Tongji Hospital affiliated to Tongji Medical College Huazhong University of Science and Technology from 11 February to 29 March 2020 were recruited for this study. The recurrent cases were considered to be mild to moderate when admitted for the second time. The recurrence group had 17 (38.64%), and the control group had 15 (46.88%) male patients (Table 1). The gender distribution did not show statistical difference (*χ*^2^ = 0.516, *P* = 0.473). The mean ages of recurrent and control cases were 49.68 ± 16.80 and 51.12 ± 15.02 years, which was not significantly different from the control cases (*t* = −0.386, *P* = 0.700). No significant difference was observed in BMI between recurrent cases and control cases (24.10 ± 3.43 *vs.* 23.98 ± 3.20, *P* = 0.911).

**Table 1.** Basic clinical and demographic characteristics of recurrent cases and control cases

Characteristics	Recurrent cases (*n* = 44)	Control cases (*n* = 32)	*t* or *χ*^2^	*P* values
Sex (male, %)	17 (38.64%)	15 (46.88%)	0.516	0.473
Age	49.68 ± 16.80	51.12 ± 15.02	−0.386	0.700
BMI	24.10 ± 3.43	23.98 ± 3.20	0.113	0.911
Hospitalisation duration	13.58 ± 4.03	15.25 ± 7.02	−1.205	0.234
Comorbidity	23 (52.27%)	11 (34.38%)	2.4	0.121
⩾2 comorbidities	5 (11.36%)	3 (9.38%)		
Hypertension	12 (27.27%)	4 (12.50%)		
Diabetes mellitus	4 (9.09%)	4 (12.50%)		
Cardiovascular disease	3 (6.82%)	0 (0.00%)		
Chronic lung diseases	3 (6.82%)	2 (6.25%)		
Hyperlipidaemia	2 (4.55%)	0 (0.00%)		
Urinary system disease	1 (2.27%)	2 (6.25%)		
Gastrointestinal diseases	1 (2.27%)	1 (3.13%)		
HBsAg positivity	1 (2.27%)	0 (0.00%)		
Thrombocytopenia	1 (2.27%)	0 (0.00%)		
Hypothyroidism	1 (2.27%)	0 (0.00%)		
Arthrolithiasis	1 (2.27%)	0 (0.00%)		
Fever	28 (63.64%)	25 (78.13%)	1.843	0.175
Cough	27 (61.36%)	21 (65.63%)	0.145	0.704
Fatigue	8 (18.18%)	12 (37.50%)	3.566	0.059
Myalgia	6 (13.64%)	6 (18.75%)	0.364	0.546
Shortness of breath	17 (38.64%)	11 (34.38%)	0.145	0.704
Dizziness	3 (6.82%)	2 (6.25%)	0.01	0.921
Gastrointestinal symptoms	9 (20.45%)	9 (28.13%)	0.603	0.437
Poor appetite	6 (13.64%)	8 (25.00%)		
Diarrhoea	4 (9.09%)	4 (12.50%)		
Nausea	5 (11.36%)	6 (18.75%)		
Vomiting	3 (6.82%)	0 (0.00%)		

HBsAg, hepatitis B virus surface antigen.

Overall, at least one concomitant disease was observed in 23 (52.27%) recurrent cases and 11 (34.38%) control cases, however, the difference was not statistically significant (*χ*^2^ = 2.4, *P* = 0.121). Hypertension (12, 27.27%) was the most frequent comorbidity observed in recurrent cases. Other relatively common concomitant diseases were diabetes mellitus (4, 9.09%), cardiovascular disease (3, 6.82%), chronic lung diseases (3, 6.82%) and hyperlipidaemia (2, 4.55%). Diabetes mellitus (4, 12.50%) and hypertension (4, 12.50%) being more common than other comorbidities (chronic lung diseases, urinary system disease and gastrointestinal diseases). Some of the rare comorbidities observed among recurrent cases are urinary system disease, gastrointestinal diseases, HBsAg positivity, thrombocytopenia, hypothyroidism and arthrolithiasis.

Among both, fever and cough were the most prevalent clinical symptoms observed in recurrent cases (28 (63.64%), 27 (61.36%)) and control cases (25 (78.13%), 21 (65.63%)). Other common symptoms at onset included shortness of breath (17, 38.64%), fatigue (8, 18.18%) and gastrointestinal symptoms (9, 20.45%), along with poor appetite, diarrhoea, nausea and vomiting. Less prevalent symptoms included myalgia (6 (13.64%) *vs.* 6 (18.75%)) and dizziness (3 (6.82%) *vs.* 2 (6.25%)).

### Laboratory parameters of recurrent cases and control cases

Elevated concentrations of ALT (35.19 ± 37.99 *vs.* 20.16 ± 12.79, *t* = 2.393, *P* = 0.020) and AST (25.12 ± 18.35 *vs.* 16.88 ± 4.46, *t* = 2.805, *P* = 0.007) were observed in recurrent cases when compared with controls. Abnormal concentrations of ALT (>33 U/l) were observed in 13 (13/42, 30.95%, 35–218 U/l) recurrent cases and 3 (3/32, 9.38%, 42–62 U/l) control cases (Table 2). Subgroup analysis showed mild elevated concentrations of ALT within 1–3 × ULN in recurrent patients (11/13, 84.62%), and moderate abnormal concentrations of ALT higher than 3 × ULN were seen only in few (2/13, 15.38%) patients. All of the three control cases had mild elevated concentrations of ALT within 1–3 × ULN. A noticeable difference in increase in concentrations of AST (6/42, 14.29%, 33–118 U/l *vs.* 0, 00%, *P* = 0.033) was observed. Of six recurrent cases, five (83.33%) had mild elevated concentrations of AST within 1–3 × ULN at subgroup analysis, and one (16.67%) had moderate abnormal concentrations of AST higher than 3 × ULN. Control cases did not show abnormal concentrations of AST. However, the difference in the increase in serum ALP and GGT levels between the two groups did not reach significance (both *P* > 0.05). Mild elevated serum ALP and GGT levels within 1–3 × ULN were seen in all cases. In addition, no significant difference was observed in the concentrations of ALB, CK, LDH, BUN, Scr and eGFR between recurrent cases and control cases (all *P* > 0.05, Table 3).

**Table 2.** Comparison of liver function transaminase of recurrent cases and control cases

Aminotransferase (*N*%)	Recurrent cases （*n* = 44）	Control cases (*n* = 32)	*χ*^2^	*P*
ALT	13 (30.95%)	3 (9.38%)	4.990	0.025*
⩽33 U/l	29 (69.05%)	29 (90.63%)		
33<*N*⩽99 U/l	11 (26.19%)	3 (9.38%)		
>99 U/l	2 (4.76%)	0 (0.00%)		
AST	6 (14.29%)	0 (0.00%)	–	0.033*
⩽32 U/l	36 (85.71%)	32 (100.00%)		
32<*N*⩽96 U/l	5 (11.91%)	0 (0.00%)		
>96 U/l	1 (2.38%)	0 (0.00%)		
ALP	1 (2.38%)	0 (0.00%)	–	1.000
⩽105 U/l	41 (97.62%)	32 (100.00%)		
105<*N*⩽315 U/l	1 (2.38%)	0 (0.00%)		
GGT	11 (26.19%)	8 (25.00%)	0.013	0.908
⩽42 U/l	31 (73.81%)	24 (75.00%)		
42<*N*⩽126 U/l	11 (26.19%)	8 (25.00%)		

ALT, alanine aminotransferase; AST, aspartate aminotransferase; ALP, alkaline phosphatase; GGT, *γ*-glutamyltransferase; *P < 0.05.

**Table 3.** Laboratory parameters of recurrent cases and control cases

Laboratory parameters (normal range)	Recurrent cases （*n* = 44）	Control cases (*n* = 32)	*t* or *χ^2^*	*P*
Immunoglobulin M (⩽10 Au/ml)	66.72 ± 117.15	54.83 ± 64.37	0.508	0.613
Immunoglobulin G (⩽10 Au/ml)	191.64 ± 108.62	164.15 ± 76.47	1.250	0.215
Albumin (35–52 g/l)	42.65 ± 3.57	40.92 ± 4.67	1.807	0.075
Alanine aminotransferase (⩽33 U/l)	35.19 ± 37.99	20.16 ± 12.79	2.393	0.020*
Aspartate aminotransferase (⩽32 U/l)	25.12 ± 18.35	16.88 ± 4.46	2.805	0.007*
Alkaline phosphatase (35–105 U/l)	66.69 ± 16.88	65.02 ± 14.07	0.449	0.654
*γ*-Glutamyltransferase (6–42 U/l)	33.10 ± 17.76	29.12 ± 17.26	0.964	0.338
Creatine kinase (⩽170 U/l)	61.32 ± 17.24	59.45 ± 31.24	0.255	0.800
Lactate dehydrogenase (135–214 U/l)	183.81 ± 34.21	177.97 ± 24.10	0.822	0.414
Urea nitrogen (3.1–8.8 mmol/l)	3.97 ± 0.87	3.93 ± 1.08	0.171	0.864
Serum creatinine (45–84 μmol/l)	63.62 ± 13.37	69.94 ± 16.67	−1.809	0.075
estimated Glomerular filtration rate (>90 ml/min)	103.29 ± 15.32	96.15 ± 16.43	1.910	0.060
Interleukin-2R (223–710 U/ml)	350.20 ± 137.37	406.56 ± 174.73	−1.544	0.127
Interleukin-6 (<7 pg/ml)	2.99 ± 3.16	5.52 ± 10.23	−1.350	0.185
Interleukin-8 (<62 pg/ml)	11.52 ± 8.09	11.79 ± 10.03	−0.129	0.898
Tumour necrosis factor-*α* (<8.1 pg/ml)	8.42 ± 3.50	7.01 ± 2.43	1.944	0.056
White blood cell count (3.5–9.510^9^/l)	6.37 ± 1.44	5.92 ± 1.14	1.460	0.148
Neutrophils count (1.8–6.3 × 10^9^/l)	3.71 ± 1.09	3.54 ± 1.03	0.692	0.491
Neutrophils ratio (40–75%)	54.34 ± 12.48	59.12 ± 7.44	−1.931	0.057
Lymphocyte count (1.1–3.2 × 10^9/l)	1.93 ± 0.57	1.71 ± 0.38	1.968	0.053
Lymphocyte ratio (20–50%)	29.77 ± 8.17	29.37 ± 6.11	0.226	0.821
Eosinophils count (0.02–0.52 × 10^9^/l)	0.18 ± 0.14	0.17 ± 0.18	0.283	0.778
Eosinophils ratio (0.4–8.0%)	2.51 ± 1.96	2.53 ± 2.11	−0.049	0.961
Haemoglobin (115–150 g/l)	135.16 ± 12.53	126.88 ± 13.44	2.759	0.007*
Platelets count (125–350 × 10^9/l)	222.00 ± 55.95	254.03 ± 49.66	−2.582	0.012*
C-reactive protein (mg/l)	3.45 ± 6.15	4.06 ± 9.30	−0.330	0.743
Procalcitonin (<0.05 ng/ml)	0.09 ± 0.12	0.06 ± 0.16	1.522	0.139
Prothrombin time (11.5–14.5 s)	13.22 ± 0.58	13.42 ± 0.48	−1.528	0.131
Activated prothrombin time (29–42 s)	36.98 ± 2.71	38.55 ± 3.72	−2.114	0.038*
D-Dimer(<0.5 ug/ml)	0.38 ± 0.42	0.38 ± 0.31	−0.008	0.993
N-terminal pro-brain natriuretic peptide (<247 pg/ml)	66.49 ± 77.23	73.27 ± 94.14	−0.313	0.755
Myoglobin (⩽106 ng/ml)	30.84 ± 9.30	38.31 ± 35.14	−1.153	0.257
Creatine kinase myoglobin (⩽3.4 ng/ml)	0.75 ± 0.49	0.82 ± 0.78	−0.517	0.607
Total lymphocyte T (CD3 + CD19-)(955–2860/ul)	1366.71 ± 298.80	1239.13 ± 477.91	0.855	0.400
Total lymphocyte B (CD3-CD19 + )(90–560/ul)	268.71 ± 84.91	239.73 ± 104.08	0.818	0.420
Helper/induced lymphocyte T (CD3 + CD4 + )(550-1440/ul)	855.71 ± 249.88	719.00 ± 273.98	1.401	0.173
Suppressor/cytotoxic lymphocyte T (CD3 + CD8 + )(320–1250/ul)	441.00 ± 124.39	451.33 ± 241.55	−0.143	0.887
Natural killer cell(CD3–/CD16 + CD56 + )(150–1100/ul)	361.07 ± 209.90	190.93 ± 99.50	2.821	0.009*
Lymphocyte T + lymphocyte B + natural killer cell(/ul)	1996.50 ± 374.94	1669.80 ± 547.61	1.861	0.074

*p < 0.05.

Concentrations of NCP virus-specific IgM and IgG were observed to be higher in recurrent cases compared to control cases (Table 3) but were not statistically significant (*t* = 0.508, *P* = 0.613, *t* = 1.250, *P* = 0.215). Interestingly, concentrations of platelet (PLT) counts were observed to be lower in recurrent cases than that in control cases (222.00 ± 55.95 × 10^9^/l *vs.* 254.03 ± 49.66 × 10^9^/l, *t* = −2.582, *P* = 0.012). However, only one patient belonging to the recurrent group represented thrombocytopenia (PLT count: 52 × 10^9^/l < 100 × 10^9^/l). In addition, no significant differences (all *P* > 0.05) were observed in both groups regarding other blood cell count (white blood cell, neutrophil, eosinophil, lymphocyte). Similarly, no significant differences (all *P* > 0.05) in concentrations of IL-2-receptor, IL-6, IL-8 and TNF-*α* between the two groups were observed. Of cases with available data, differences regarding coagulation parameters, myocardial markers, N-terminal pro-brain natriuretic peptide concentrations and lymphocyte were considered non-significant (all *P* > 0.05). In addition, the pulmonary imaging indicated that the recurrent cases were observed to be in the absorptive stage of viral infection.

In addition, multivariate analysis showed that the predictive value of elevated concentrations of ALT for the risk of ‘recurrence’ of COVID-19 did not reach statistical significance (OR = 1.040, 95% confidence interval 0.985–1.099, *P* = 0.157, Table 4). Delightingly, multivariate analysis showed that elevated concentrations of AST may be recognised as an independent risk factor of ‘recurrence’ of COVID-19 (OR = 1.175, 95% confidence interval 1.002–1.379, *P* = 0.047, Table 5).

**Table 4.** Multivariate analysis of the ALT prediction of ‘recurrence’ of COVID-19

	B	s.e.	Wald	OR	95% CI	*P*
ALT	0.039	0.028	2.001	1.040	0.985–1.099	0.157
BMI	−0.009	0.108	0.007	0.991	0.802–1.225	0.933
Haemoglobin	−0.012	0.035	0.118	0.988	0.922–1.058	0.731
PLT count	−0.027	0.012	4.931	0.973	0.950–0.997	0.026
Activated prothrombin time	−0.098	0.118	0.694	0.907	0.720–1.142	0.405
Constant	11.454	7.491	2.338			

ALT, alanine aminotransferase; PLT, platelets; 95% CI, 95% confidence interval.

**Table 5.** Multivariate analysis of the AST prediction of ‘recurrence’ of COVID-19

	B	s.e.	Wald	OR	95% CI	*P*
AST	0.162	0.081	3.935	1.175	1.002–1.379	0.047
BMI	0.050	0.117	0.178	1.051	0.835–1.323	0.673
Haemoglobin	−0.014	0.033	0.179	0.986	0.924–1.052	0.672
PLT count	−0.029	0.013	5.351	0.971	0.947–0.996	0.021
Activated prothrombin time	−0.107	0.122	0.769	0.899	0.708–1.141	0.381
Constant	8.997	7.359	1.495			

AST, aspartate aminotransferase; PLT, platelets; 95% CI, 95% confidence interval.

## Discussion

This study retrospectively reviewed the clinical and demographic characteristics and laboratory parameters of the recurrent cases of COVID-19. Various concomitant diseases and markedly elevated concentrations of ALT and AST were observed in recurrent cases of COVID-19 than those in control cases. Fever and cough were the most prevalent clinical symptoms observed. This study data showed that elevated concentrations of ALT and AST may predict the risk of ‘recurrence’ of COVID-19, which may provide insights into the prevention and control of COVID-19 in the future.

Concerns were aroused on the ‘recurrence’ of disease in patients who had recovered from COVID-19 infections that weather these cases still may be SARS-CoV-2 carriers and needed additional monitoring, isolation and precautions. It is also observed that some COVID-19 recovery cases may experience a fluctuating period between the relief of clinical symptoms or pulmonary imaging and real recovery from SARS-CoV-2 infections. Some studies have reported that the clinical features and the potential risk factors of SARS-CoV-2 turned positive in patients after they were discharged [7–17]. The reported recurrence rate ranged from 9.09% to 21.43% [7, 12, 16]. This study is one of the studies that recruited the largest size of recurrent cases of COVID-19 till to now. All the 44 recurrent patients from COVID-19 infections, who were recruited for this study, met the discharge criteria while they showed positive for nucleic acid tests post isolation. None of these cases showed aggravating clinical symptoms, which was similar to the results demonstrated by Yuan *et al.* [12]. Recent studies reported that fever and cough were the main clinical symptoms observed in COVID-19 patients, who tested positive after discharge [11–13, 15], which is similar to the results obtained in our study (fever (28, 63.64% *vs.* 25, 78.13%) and cough (27, 61.36% *vs.* 21, 65.63%)). However, the differences were not statistically significant. Moreover, recurrent patients in this study represented more concomitant diseases (23, 52.27% *vs.* 11, 34.38%), no matter in the number of patients with comorbidities or in the types of comorbidities. Among them, the most prevalent comorbidity was hypertension. These results were similar to the results by Zhou *et al*., which suggested that COVID-19 patients suffering from the basic concomitant disease may have a higher risk of recurrence [18]. However, to further evaluate the potential significance, a larger sample of discharged COVID-19 patients with a longer follow-up period was needed.

Notably, along with respiratory symptoms, including fever, dry cough and dyspnoea primarily [6], COVID-19 patients also presented some cases of varying degrees of abnormal liver enzymes throughout the disease [19–25]. Wang and coworkers summarised that approximately 14–51% of COVID-19 patients had abnormal concentrations of ALT and AST [26]. The study by Ning and coworkers reported that 52% of deceased patients with COVID-19 represented abnormal serum AST levels (>40 U/l), indicating that severe COVID-19 patients tend to have higher rates of abnormal liver enzymes [25]. This result was in agreement with a prospective study performed by Huang *et al*. [27] and a recent systematic review about COVID-19 and liver function conducted by Kukla *et al.* [24]. Abnormal liver enzymes in patients with COVID-19 were demonstrated by some potential mechanisms. First is the SARS-CoV-2 viral infection directly on liver cells. Previous studies have demonstrated that SARS-CoV-2 may directly target the angiotensin-converting enzyme 2 (ACE2)-positive cholangiocytes, which induces hepatic injury [28, 29]. The most recent study in *J Hepatol* by Zhao and coworkers [30] reported that numerous intact coronavirus particles ranging between 70 and 120 nm in the cytoplasm of hepatocytes were observed by the transmission electron microscope, suggesting a direct cytopathic lesion on liver cells caused by SARS-CoV-2 infection. These findings indicated that direct SARS-CoV-2 infection was a crucial cause of COVID-19-related abnormal liver enzymes. In addition, other potential causes were cytokine storm-associated systemic inflammatory response syndrome, hyperactivated immune responses, psychological stress, hepatic ischemia, and hypoxia-reperfusion dysfunction, progression of pre-existing liver diseases and drug-induced liver injury [16, 31–34].

Interestingly, elevated concentrations of ALT and AST in recurrent cases compared with the control cases (*t*_ALT_ = 2.393, *P* = 0.020; *t*_AST_ = 2.805, *P* = 0.007) were shown. In addition, mild to moderate abnormal concentrations of ALT and AST in recurrent cases were observed in the subgroup analysis. Delightingly, multivariate analysis showed that elevated concentrations of AST may be recognised as an independent risk factor of ‘recurrence’ of COVID-19. However, multivariate analysis showed that the predictive value of elevated concentrations of ALT for the risk of ‘recurrence’ of COVID-19 did not reach statistical significance. These results indicated that ‘recurrence’ of COVID-19 may be a complex disease process with multiple factors. However, the study by Li *et al*. summarised that all recruited 13 discharged patients from COVID-19 infections had normal transaminase concentrations [15]. Subsequently, Ye *et al*. reported that all five recurrent COVID-19 patients had normal ALT and AST serum levels [7]. Similarly, a recent retrospective study showed that one patient admitted for the second time with positive RT-PCR test of SARS-CoV-2 after discharge represented elevated serum levels of ALT and AST [11]. More importantly, except for one recurrence case with HBsAg positivity, the majority of the recruited cases had no basic liver diseases. Our data suggested that elevated concentrations of ALT and AST may be recognised as predictive markers for the ‘recurrence’ of COVID-19. The most recent study in *Hepatology* by Hundt *et al*. in 29 July 2020 reported that abnormal liver tests were commonly observed in hospitalised patients with COVID-19, both at admission (AST 66.9%, ALT 41.6%) and peak hospitalisation (AST 83.4%, ALT 61.6%) [35]. In addition, multivariate analysis revealed an association between abnormal liver tests and severe COVID-19, including ICU admission, mechanical ventilation and death, which suggested that abnormal liver tests may be associated with poorer clinical outcomes, which suggested that abnormal liver tests may be associated with poorer clinical outcomes [35]. These data further demonstrated the predictive value of elevated concentrations of ALT and AST in the progression and prognosis, as well as the ‘recurrence’ of COVID-19. In addition, there is no evidence to clarify whether these readmitted COVID-19 patients with positive RT-PCR test of SARS-CoV-2 were infectious, or they became chronic SARS-CoV-2 carriers in the future. All the recurrent cases in this study were managed based on the principle of prevention and treatment of COVID-19. To our delight, all the recurrent patients reached the standard of symptom relief and discharge criteria according to the diagnosis and treatment protocol for NCP (trial version 7) released by the National Health Commission and National Administration of Traditional Chinese Medicine. However, there are several limitations to our study. The recovery rate could not be calculated in this study due to: (i) first admissions of some recurrent patients were not in Guanggu Campus of Tongji Hospital; (ii) patients not discharged from different hospitals were integrated at the end of March according to the unified arrangement of ‘Wuhan and Hubei defense Wars’. Some severe cases and some patients who were not discharged from the Guanggu Campus of Tongji Hospital were transferred to the Zhongfaxincheng Campus of Tongji Hospital.

In conclusion, this study showed the clinical and laboratory characteristics of recurrent cases of COVID-19. More importantly, our study results suggested that elevated concentrations of ALT and AST may be recognised as predictive markers for the risk of ‘recurrence’ of COVID-19, which, in turn, may provide certain insights into the prevention and control of this infectious and fatal disease in the future. To further understand the progression and prognosis of COVID-19, a larger cohort of recovery COVID-19 patients with a longer and standard follow-up period was needed, such that the recurrence rate can be reduced.

## Data Availability

All data generated or analysed during this study are included in this article.
